# The role of foliar endophytes in modulating southern corn rust severity: implications for biocontrol strategies

**DOI:** 10.3389/fpls.2025.1554915

**Published:** 2025-05-23

**Authors:** Lujia Yang, Lili Li, Yingying Song, Yongsheng Zhang, Jie Yang, Hongying Cui, Wenxiu Guo, Suhong Lv, Xingyuan Men

**Affiliations:** ^1^ Shandong Key Laboratory for Green Prevention and Control of Agricultural Pests, Institute of Plant Protection, Shandong Academy of Agricultural Science, Jinan, China; ^2^ College of Plant Protection, Hunan Agricultural University, Changsha, China

**Keywords:** southern corn rust, *Puccina ploysora*, foliar endophyte fungi, microbial diversity, host-pathogen interactions

## Abstract

Southern corn rust (SCR), caused by *Puccinia polysora*, is a major foliar disease that threatens global maize production. Current SCR management strategies prioritize genetic resistance and chemical control, but how foliar endophytic fungal communities modulate host susceptibility to *P. polysora* remains poorly understood. In this study, we profiled the endophytic communities in *P. polysora*-infected and non-infected maize leaves across 14 geographically distinct regions in eastern China. Our results revealed that *P. polysora* infection significantly altered the foliar endophytic community, with infected leaves exhibiting higher operational taxonomic unit (OTU) richness (722 vs. 572 OTUs) while reducing community evenness. Diversity metrics were significantly altered, with significant reductions in Shannon diversity and Chao1 index values for non-infected states. Network analysis revealed that infection caused a notable reduction in microbial connectivity and complexity, particularly in low- and medium-susceptibility regions, where positive intertaxon associations declined by 42.6% and 35.3%, respectively. High-susceptibility region networks retained greater stability, suggesting differential microbial resilience under pathogen pressure. Redundancy analysis further demonstrated that temperature was the dominant environmental factor shaping microbial assemblages, especially under infection conditions. Notably, correlation analysis further revealed that *Alternaria* was positively associated with host resistance (*r* = 0.37, *p* = 0.05), underscoring its potential role in enhancing resistance to *P. polysora*. Conversely, *Dioszegia* and *Naganishia* were negatively correlated with resistance (*r* = −0.36, *p* = 0.056; and *r* = −0.34, *p* = 0.074, respectively), implying potential roles in facilitating infection. This study reveals key mechanistic links between foliar endophytic communities and SCR infection, providing a basis for sustainable biocontrol interventions in maize.

## 1 Introduction

Maize (*Zea mays* L.) is a globally vital crop that is integral to food security and economic stability. In China, the increasing demand for maize has prompted efforts to develop high-yielding and disease-resistant cultivars, contributing to sustained production gains. However, maize cultivation continues to face substantial threats from both biotic and abiotic stressors. Among these is southern corn rust (SCR), caused by the fungal pathogen *Puccinia polysora* Underw. (*P. polysora*), which has emerged as a major constraint, particularly in subtropical and temperate maize-growing regions (Sun et al., 2021a; Sun et al., 2023; Yang et al., 2024). Globally, SCR can cause yield losses ranging from 10% to 50% in susceptible cultivars. In 2024, SCR was responsible for an estimated loss of 244.27 million bushels of corn in the USA alone (Crop Protection Network, 2025). Since its first report in China in 1972, SCR has expanded swiftly across major maize-growing regions, now affecting more than 100 million acres annually, with approximately 19.38 million hectares subjected to chemical control measures (Ma et al., 2022; Ministry of Agriculture and Rural Affairs of the People’s Republic of China, 2023). In epidemic years, yield losses can reach 50%, and their widespread distribution threatens national food security (Wang et al., 2020; Meng et al., 2020; Sun et al., 2021a; Ma et al., 2022).

The geographic spread of SCR is coupled with the rapid dissemination and capacity of the pathogen to inflict severe crop damage. While fungicides provide short-term control of SCR, their widespread application poses significant challenges, including environmental contamination, increased production costs, and the emergence of fungicide-resistant *P. polysora* strains (Ma et al., 2022; Yang et al., 2024). These concerns have intensified interest in sustainable alternatives to fungicide control. Among these, microbial-based strategies have gained attention for their ecological compatibility and potential long-term efficacy.

Foliar endophytes play a pivotal role in modulating plant health by influencing host immune responses, outcompeting pathogens for niche occupancy, and altering the leaf microenvironment to suppress disease development (Busby et al., 2013; Hardoim et al., 2015; Busby et al., 2016; Collinge et al., 2022). Community-level shifts in foliar microbiota have been shown to significantly affect disease susceptibility (Johnston-Monje and Raizada, 2011; Raghavendra and Newcombe, 2013; Busby et al., 2016; Collinge et al., 2022). A study revealed that *Exserohilum turcicum*, the causal agent of northern corn leaf blight, is associated with reduced microbial diversity, potentially compromising host resilience. This association underscores the broader role of microbial community composition in modulating plant health (Chao et al., 2025). Studies have also shown that certain *Bacillus* and *Pseudomonas* species isolated from maize leaves have antagonistic effects on *Puccinia sorghi* while simultaneously promoting systemic resistance (Sartori et al., 2017). These findings highlight the potential of harnessing the native microbiome as a sustainable strategy for enhancing crop disease resistance and reducing dependence on chemical control.

While previous studies have largely focused on endophyte–pathogen interactions under controlled conditions (Porras-Alfaro and Bayman, 2011; Liu et al., 2022), the extent to which these relationships are shaped by natural field variability remains unclear. Emerging evidence suggests that geographic variations in disease severity may have a stronger influence on the composition and functional dynamics of foliar endophytic fungal communities than pathogen–host interactions alone (Wheeler et al., 2019; Wagner et al., 2020; Tedersoo et al., 2020a, b; Tay et al., 2023; Wang et al., 2023). However, investigations into how these communities respond to *P. polysora* under natural infection pressure are notably lacking. Moreover, the role of spatial heterogeneity in modulating endophyte assemblages and its correlation with disease outcomes has not been systematically addressed. These gaps constrain the development of microbiome-informed strategies for SCR suppression and limit our capacity to harness beneficial endophytes in field-based disease management.

Given that pathogen-induced shifts in foliar endophytic fungal communities may directly influence disease trajectories, addressing these gaps represents a critical frontier in plant–microbe interaction research. A mechanistic understanding of the assembly, composition, and functional traits, particularly in the context of *P. polysora* infection, is essential for linking microbial diversity to host health and disease resistance in maize. Realizing the potential of such approaches requires detailed insights into the structure and ecological roles of endophytic communities in both infected and non-infected tissues and how these roles are related to gradients of disease severity.

This study aims to elucidate the ecological dynamics of foliar endophytic fungal communities in maize under natural *P. polysora* infection and to determine how shifts in community composition correlate with disease severity. We hypothesize that common endophytic fungi present in both healthy and *P. polysora*-infected maize leaves influence SCR severity through shifts in community composition and functional interactions. To test this hypothesis, we i) characterize the taxonomic composition, richness, and diversity of foliar endophytic communities in healthy versus infected leaves using high-throughput internal transcribed spacer (ITS) amplicon sequencing; ii) examine the relationships between community structure and the relative disease index (RDI) across a gradient of field infection severity; iii) identify keystone taxa and ecological interactions using co-occurrence network analysis and random forest modeling; and iv) assess the role of environmental drivers, such as temperature, humidity, and rainfall, in shaping endophytic community assembly and disease dynamics. By identifying key fungal taxa that potentially suppress or promote SCR, we provide a framework for developing sustainable, microbiome-informed biocontrol strategies.

## 2 Materials and methods

### 2.1 Study locations, maize material collection, and disease assessment

**Figure 1:**
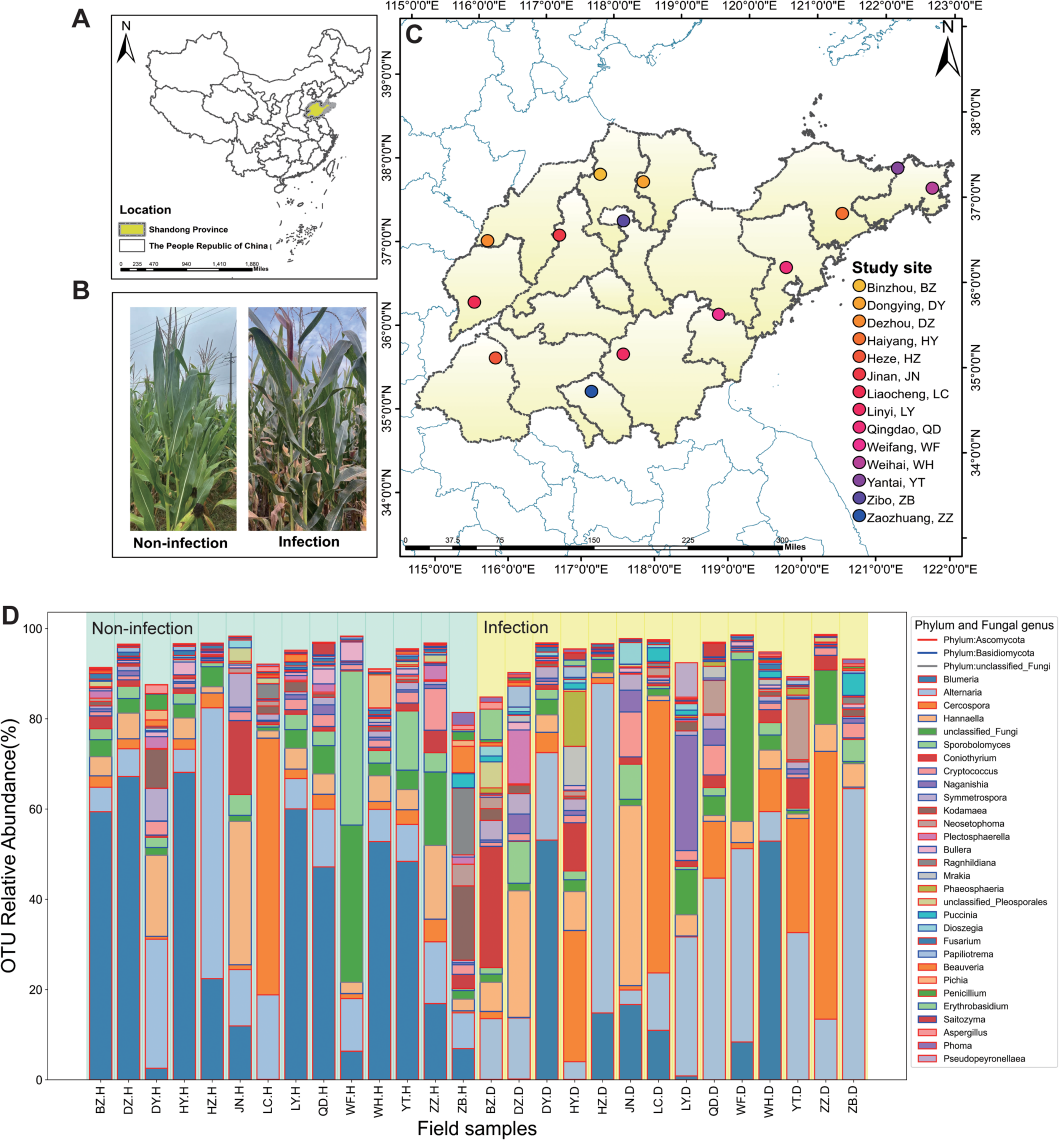
Foliar endophytes across the study location are compositionally variable. **(A)** The location of Shandong Province in China. **(B)** Non-infection (H) and infection (D) symptoms of southern corn rust (SCR) of maize in the field. **(C)** Multiple sampling sites in Shandong Province. **(D)** The operational taxonomic unit (OTU) relative abundance within non-infected and *Puccinia polysora*-infected samples at the phylum level and genus level are shown for taxa with >1% mean relative abundance across all sites. Field samples are grouped along the x-axis by study, with color-coded site labels located above the bars. The lines represent different phyla, and the different colored columns represent the taxonomic units of different fungal genera.

Maize plants were sampled and assessed during the 38th to 40th weeks of the year (the grain-filling stage) across 14 geographically distinct regions in Shandong Province, China (**Figures 1A, C**) (Yang et al., 2024). At each sampling site, six leaves were collected from healthy maize plants, alongside six leaves from *P. polysora*-infected plants. Infected leaves were selected based on lesion coverage of 40%–60% of the leaf area with *P. polysora* pustules (**Figure 1B**). All samples were taken and assessed from the fourth leaf up from the base of each plant (Yang et al., 2024). The samples were immediately placed in sterile bags and transported to the laboratory on dry ice to preserve microbial integrity.

#### 2.1.1 Endophyte sample collection

To eliminate epiphytic microorganisms and remove *P. polysora* pustules from the leaf surface, a rigorous sterilization and washing protocol was applied. Approximately 2 g of plant tissue was excised from each sample and initially rinsed with sterile water to remove surface debris. For infected samples, leaf tissues were immersed in 1× phosphate-buffered saline (PBS) and vibrated for 1 h to facilitate the detachment of *P. polysora* uredinia into the solution. All the samples were subsequently surface sterilized by sequential immersion in 70% ethanol for 1 min and 2% sodium hypochlorite for 5 min, followed by three rinses in sterile distilled water (Yang et al., 2023). To ensure the complete removal of residual disinfectants, the tissues were additionally washed three times with 1× PBS and then air-dried under sterile conditions. The sterilized leaf tissues were then frozen at −80°C for subsequent DNA extraction.

#### 2.1.2 Disease and regional susceptibility assessment

For disease assessment, *P. polysora*-infected maize leaves were evaluated once at each sampling site during the same collection period. A 5-point survey method was employed, in which 10 maize plants per site were assessed. Evaluations began from the fourth leaf above the base of each plant. The number of infected leaves and the severity of infection were visually recorded. Disease severity was classified on the basis of the percentage of leaf area covered by *P. polysora* pustules, using the following scale: level 0 (0%), level 1 (1%–5%), level 3 (6%–25%), level 5 (26%–50%), level 7 (51%–75%), and level 9 (76%–100%) (Yang et al., 2024). The disease index (DI) (Equation 1) values obtained from each assessed site depict the severity of the SCR (Madden et al., 2017; Yang et al., 2023):


(1) $$\text{Disease index DI\%}=\sum \frac{\left(\text{No}.\text{ of diseased leaves }\times \text{ disease grade per leaf}\right)}{\text{Total no}.\text{ of disease leaves }\times \text{ highest disease grade}}\;\times 100\;\;$$

The RDI (Equation 2) was computed to normalize SCR susceptibility across regions (Xu and Hu, 2015):


(2) $$\text{Relative disease index }\left(\text{RDI}\right)=1-\frac{\text{The DI of each study site}}{\text{The highest DI of all study sites}}$$

The regions were categorized on the basis of RDI values (0–1) as follows: high-susceptibility (HS) regions, RDI < 0.20; medium-susceptibility (MS) regions, 0.20 ≤ RDI < 0.50; low-susceptibility (LS) regions, 0.50 ≤ RDI < 0.80; and high-resistance (HR) regions, 0.80 ≤ RDI ≤ 1.00.

### 2.2 DNA extraction and high-throughput sequencing protocol

Total genomic DNA was extracted from maize leaf tissues via a modified cetyltrimethylammonium bromide (CTAB) protocol (Yang et al., 2023). Leaf samples were transferred into 2-mL microcentrifuge tubes and ground in liquid nitrogen. Preheated 2% CTAB extraction buffer was then added, and the mixture was incubated at 65°C for 1 h with random mixing. Following incubation, an equal volume of phenol to chloroform:isoamyl alcohol (24:1) was added to each tube, and the samples were centrifuged at 10,000 rpm for 10 min. The upper aqueous phase ensuring no visible cloudiness was transferred to new 1.5-mL tubes. DNA was precipitated by adding 0.6 volumes of isopropanol and incubating at −20°C for 1 h (Abeywickrama et al., 2023). The DNA pellet was collected by centrifugation at 12,000 rpm for 10 min, washed twice with 70% ethanol, dried under vacuum, and resuspended in 20–30 µL of Tris-EDTA (TE) buffer containing RNase. DNA concentrations were quantified via a NanoDrop 2000 spectrophotometer (Thermo Fisher Scientific, Waltham, MA, USA), and DNA quality was evaluated via 1% agarose gel electrophoresis.

High-throughput sequencing (HTS) amplicon sequencing was used to identify endophytic fungal taxa within maize leaf tissues following established protocols (Pérez-Jaramillo et al., 2018; Xia et al., 2020). Individual morphotypes were selected for molecular identification based on the amplification of the ITS region of the nuclear ribosomal DNA (rDNA). The ITS region was targeted using the primers ITS1-F and ITS2-R and subsequently used for amplicon library preparation (Smith and Peay, 2014). PCR amplification was performed in a 30-µL reaction volume containing 15 µL of 2×Hieff^®^ Robust PCR master mix (Yeasen, Shanghai, China), 1 µL each of primers (forward and reverse), 20 ng of DNA template, and nuclease-free water to the final volume. The thermal cycling conditions were as follows: initial denaturation at 94°C for 3 min; 25 cycles of denaturation at 94°C for 30 s, annealing at 55°C for 30 s, and extension at 72°C for 30 s; and a final extension at 72°C for 5 min.

Amplicons were purified using AMPure XP magnetic beads and quantified using a Qubit fluorometer, and dual-index barcodes were ligated to each sample following the standard Illumina library preparation protocol (Nilsson et al., 2019). Equimolar amounts of purified amplicons were pooled and sequenced using the Illumina MiSeq platform (Illumina, San Diego, CA, USA) with a 2 × 250 bp paired-end configuration, generating approximately 45,000 raw reads per sample. The raw sequence data were subjected to quality filtering using the QIIME2 (version 2024.2) and USEARCH pipelines. Low-quality reads with a Phred score < Q30, ambiguous bases, or read lengths < 200 bp were discarded. Paired-end reads were merged using a minimum 20-bp overlap and a maximum mismatch rate of 10%. Chimeric sequences were identified and excluded using UCHIME. After sequencing, the raw reads were quality-checked, and regions with a base error probability > 5% were trimmed using GENEIOUS 6.1.7 (Kearse et al., 2012). Paired-end reads were assembled into consensus sequences, and chimeric sequences were removed.

### 2.3 Data processing and bioinformatics analysis

#### 2.3.1 OTU clustering, taxonomic assignment, and database validation

Cleaned sequences were clustered into operational taxonomic units (OTUs) at a 97% similarity threshold using the URAPE algorithm against the UNITE fungal ITS reference database (Wagner et al., 2020). Taxonomic assignments were performed using QIIME2. OTUs with fewer than 10 reads were excluded to reduce analytical noise and enhance result robustness.

To ensure taxonomic accuracy, OTUs were annotated to the genus level only if they shared ≥95% sequence identity with a reference, whereas species-level assignments required ≥99% identity and ≥90% alignment coverage (Raja et al., 2017). For validation, a subset of high-abundance OTUs was subjected to BLAST searches against the NCBI nucleotide database (https://blast.ncbi.nlm.nih.gov/Blast.cgi). Only OTUs with consistent genus-level classification across both databases were retained for ecological interpretation.

#### 2.3.2 Diversity indices and community analysis

To investigate the influence of *P. polysora* infection on the endophytic community composition, both alpha and beta diversity indices were computed. Differential abundance analysis between non-infected and *P. polysora*-infected samples was conducted using Python (v.3.9.0). OTUs with statistically significant differences (*p* < 0.05) were identified as potential indicators of SCR susceptibility. Alpha diversity indices, including the ACE, Chao1, Shannon, and Simpson indices, were used to evaluate the richness and evenness within samples. Fungal richness and abundance were defined as the average number of OTUs and isolates per sample, respectively, and were calculated using Species Diversity and Richness v4.0 (Martins et al., 2021). Beta diversity was assessed using the Bray–Curtis and weighted UniFrac dissimilarity indices. Community composition differences between the infected and non-infected groups were visualized through principal coordinate analysis (PCoA). These analyses provide insights into how SCR infection influences the composition and diversity of maize foliar fungal endophytes under natural field conditions.

### 2.4 Statistical analysis

#### 2.4.1 Diversity and community composition

Microbial diversity differences were analyzed using an analysis of variance (ANOVA) in Python (v.3.9.0), with *post-hoc* comparisons using Tukey’s honestly significant difference (HSD) test (*p* < 0.05). To quantitatively link shifts in endophytic diversity to *P. polysora* infection, permutational multivariate analysis of variance (PERMANOVA) was employed to assess the effects of infection status and sampling site on community variance. Pearson’s correlation analysis was used to explore the relationship between endophytic diversity and SCR severity.

#### 2.4.2 Network analysis

Co-occurrence network analysis was used to identify potential synergistic or antagonistic interactions among fungal genera during *P. polysora* infection. Networks were constructed to compare microbial interactions in infected vs. non-infected samples and to detect keystone taxa that formed ecological hubs. Functional and phylogenetic structural shifts were evaluated to reveal community-level reorganization and adaptation in relation to SCR severity.

#### 2.4.3 Differential abundance and functional analysis

Redundancy analysis (RDA) was performed using the Hellinger-transformed OTU data to evaluate the effects of *P. polysora* infection and abiotic factors [temperature (T), rainfall (R), and relative humidity (RH)] on endophytic fungal communities. RDA was conducted in Python using linear regression followed by principal component analysis (PCA) of the fitted values. The statistical significance of the model and constrained axes was evaluated using permutation tests (n = 999). The relative contribution of each explanatory variable was visualized via ordination plots with fitted environmental vectors.

To identify endophytic taxa and functional pathways associated with infection, a random forest algorithm was used to identify the most critical fungal OTUs for distinguishing infected from non-infected samples, with the mean decrease in Gini impurity quantifying each OTU’s importance (Martins et al., 2021). Specific OTUs associated with *P. polysora*-infected and non-infected samples were identified, offering insights into microbial roles in disease suppression or facilitation (Hardoim et al., 2015). Fungal OTUs were categorized as commensal (no host effect), beneficial (promoting growth or protection), or pathogenic (including latent pathogens). After grouping, the changes in fungal abundance and richness of each functional group across different disease susceptibilities were determined among the LS, MS, and HS regions. Regression analysis and ANOVA were then applied to correlate the relative abundance of fungal OTUs identified by the random forest with the RDI.

## 3 Results

### 3.1 Differences in fungal community composition between non-infected and *P. polysora*-infected maize leaves

At both the OTU and genus levels, the fungal community composition differed between non-infected and *P. polysora*-infected maize leaves. Compared with non-infected leaves, infected leaves harbored a greater number of OTUs (n = 722; n = 572), reflecting an infection-induced expansion of the foliar endophytic community. The fungal communities in both groups were dominated by members of the phyla Ascomycota (non-infection = 62.76%, infection = 63.02%) and Basidiomycota (non-infection = 29.90%, infection = 32.55%). The Ascomycota phylum is composed of the key genera *Blumeria*, *Alternaria*, and *Cercospora*, whereas Basidiomycota is represented by the genera *Hannaella*, *Sporobolomyces*, and *Cryptococcus*, each displaying distinct distribution patterns under healthy and infected conditions (**Figure 1D**).

Non-infected samples were characterized by relatively uniform community structures, with dominant genera including *Bullera* (33.6%), *Alternaria* (14.62%), *Cercospora* (6.13%), *Hannaella* (7.73%), *Sporobolomyces* (5.52%), *Coniothyrium* (2.60%), *Cryptococcus* (2.13%), and *Puccinia* (0.31%) (**Figure 1D**). In contrast, infection induced a shift in fungal community structure, with increased relative abundances of *Alternaria* (26.76%), *Cercospora* (14.75%), *Hannaella* (8.30%), *Coniothyrium* (4.21%), *Cryptococcus* (2.29%), and *Puccinia* (1.01%).

At the species level, *Blumeria graminis*, *Cercospora coniogrammes*, *Hannaella sinensis*, *Naganishia albida*, and *Kodamaea ohmeri* were consistently present across multiple sites, with higher abundances observed in infected leaves compared to non-infected leaves (**Supplementary Figure S1**). While most sites presented increased fungal richness in infected leaves, the patterns were site-specific, suggesting that *P. polysora* infection may drive localized shifts in endophytic community composition and diversity.

### 3.2 Diversity analysis of endophytic fungal communities

**Figure 2:**
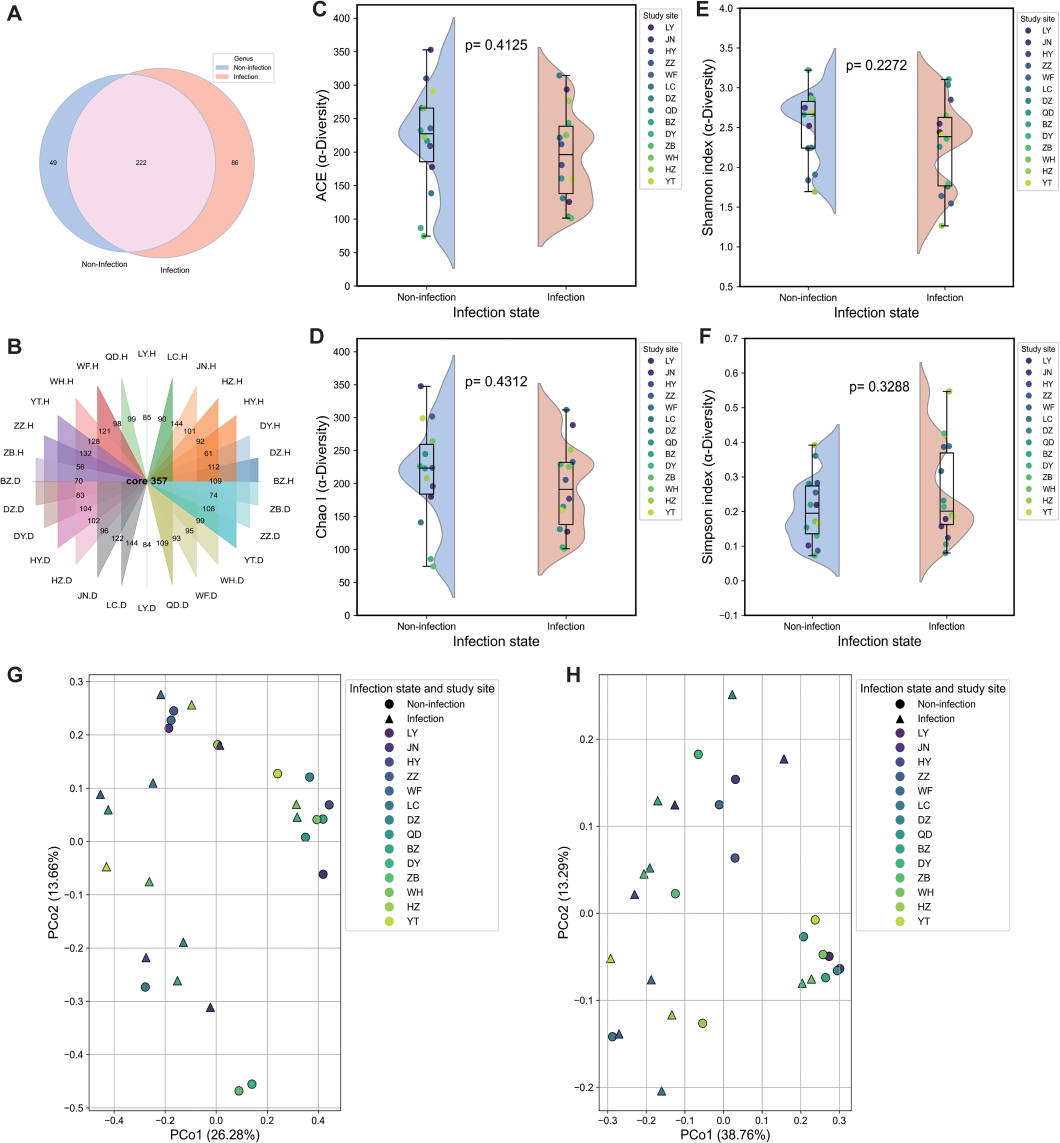
Differential analysis of fungal microbial communities and diversity. **(A)** Total analysis of genera in the non-infected and infected groups. **(B)** Analysis of genera associated with non-infected and infected states at each sampling site. **(C)** ACE index. **(D)** Chao index. **(E)** Shannon index. **(F)** Simpson index. Box plots depict medians (central horizontal lines), interquartile ranges (boxes), 95% confidence intervals (whiskers), and outliers (dots). Statistically significant differences between pairs of values are shown over horizontal lines. *p*-Values indicate statistically significant differences between the non-infected and infected samples. **(G, H)** Principal coordinate analysis (PCoA) plots based on weighted UniFrac **(G)** and Bray–Curtis **(H)** dissimilarity of non-infected and infected fungal communities at the study sites.

Comparative diversity analyses revealed substantial differences in the fungal community between the non-infected and *P. polysora*-infected groups. At the genus level, the non-infected samples contained 49 unique genera, whereas the infected samples contained 86 unique genera, with 222 genera shared between the two groups (**Figure 2A**). These findings suggest that pathogen infection may facilitate the recruitment or activation of rare or dormant fungal taxa. Analysis of fungal richness across sites revealed that 64% of the regions presented relatively high fungal richness in non-infected samples (**Figure 2B**). Alpha diversity further highlighted significant differences. Both ACE and Chao1 indices were lower in the infected samples (**Figures 2C, D**), indicating reduced species richness. In contrast, the Shannon index slightly increased fungal diversity in the infected samples, suggesting that infection increased community heterogeneity (**Figure 2E**). The Simpson index was marginally greater in non-infected samples (**Figure 2F**), reflecting greater evenness in the healthy state.

Beta diversity analysis, as visualized by PCoA, revealed distinct clustering between the non-infected and infected groups. Weighted UniFrac distances revealed that PCo1 and PCo2 explained 38.76% and 12.35% of the variance, respectively (**Figure 2G**), with 71% of the non-infected individuals forming a distinct cluster. Similar clustering patterns were observed via the Bray–Curtis dissimilarity (PCo1 = 26.28%, PCo2 = 13.65%), with 78% of the non-infected samples clustering into a single group (**Figure 2H**).

### 3.3 Foliar endophyte abundance across SCR susceptibility regions and environmental correlations

**Figure 3:**
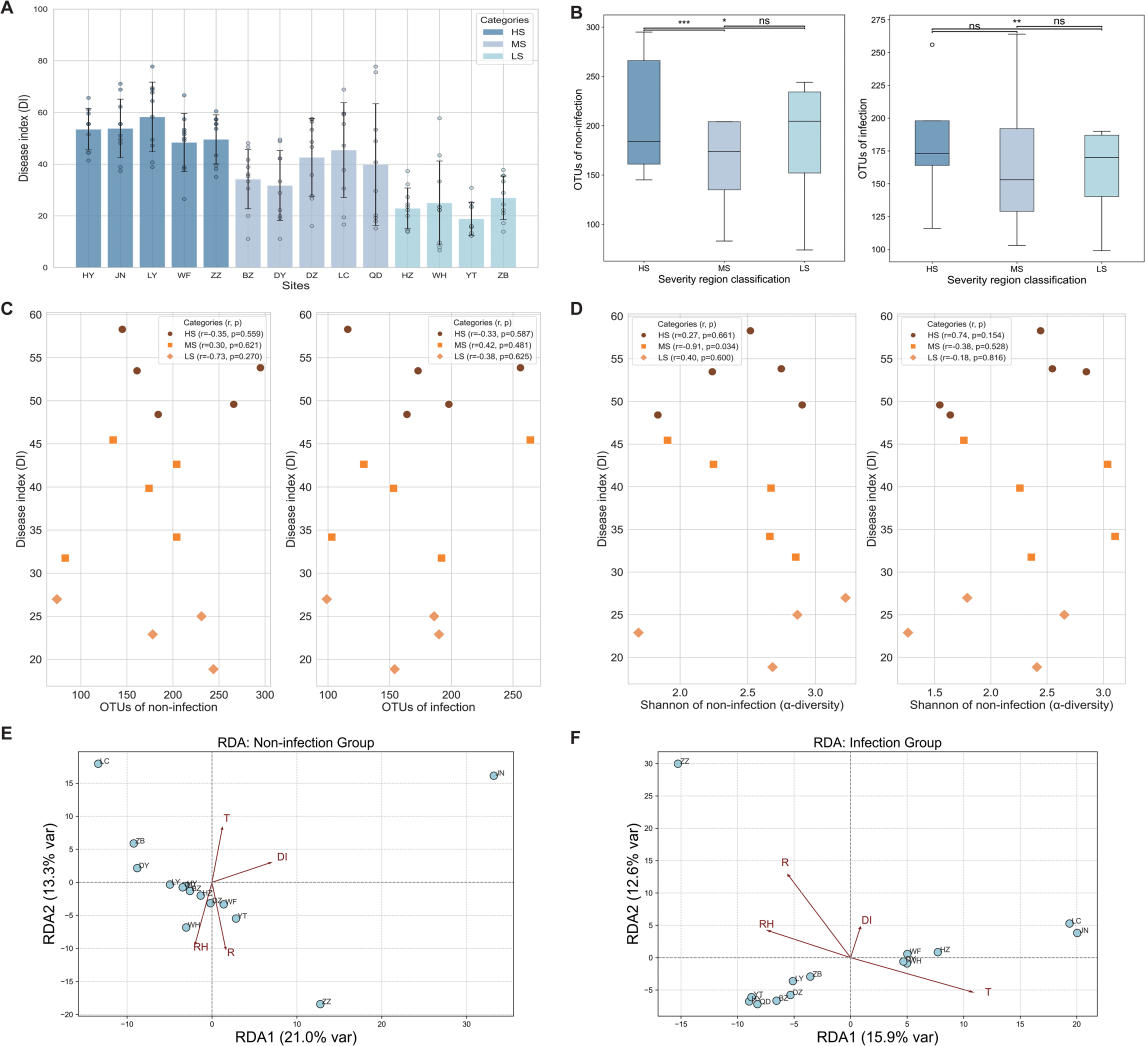
Microbial diversity and its relationship with disease severity across regions of varying susceptibility. **(A)** Disease index (DI) of southern corn rust (SCR) as the means of replicates (n = 10), displaying Standard error (SE) values across 14 study sites. **(B)** Number of operational taxonomic units (OTUs) of non-infected and infected samples observed in regions exhibiting different levels of disease severity. Box plots depict medians (central horizontal lines), interquartile ranges (boxes), 95% confidence intervals (whiskers), and outliers (dots). Statistically significant differences between pairs of values are shown over horizontal lines. Statistical significance among high susceptibility (HS), medium susceptibility (MS), and low susceptibility (LS) was evaluated using Tukey’s honestly significant difference (HSD) test, with differences deemed significant at *p < 0.05, **p < 0.01, and ***p < 0.001. **(C)** Correlations between OTUs and DI in the HS, MS, and LS regions. **(D)** Correlations between alpha diversity (Shannon index) and DI in the HS, MS, and LS regions. **(E, F)** Redundancy analysis (RDA) of foliar endophytic fungal community composition in relation to environmental variables under non-infected and *Puccinia polysora*-infected conditions. The scattered points represent different study regions, and T, RH, and R represent temperature, relative humidity, and rainfall, respectively.

The DI values clearly classified the 14 sites based on their susceptibility to SCR, revealing significant variation across the HS, MS, and LS regions (**Figure 3A**). The DI values ranged from 48.42 to 58.29 for the HS regions (HY, JN, LY, WF, and ZZ), 31.76 to 45.46 for the MS regions (BZ, DY, DZ, LC, and QD), and 18.86 to 26.98 for the LS regions (HZ, WH, YT, and ZB) (**Figure 3A**). Across all categories, *P. polysora* infection led to a significant reduction in OTU richness (**Figure 3B**). In the non-infected state, the HS and MS regions presented greater richness than the LS regions, with a significant difference observed between the MS and LS regions (*p* < 0.001 and *p* < 0.05) (**Figure 3B**). Under infection, the OTU richness was significantly greater in the HS regions than in the LS regions (*p* < 0.001), whereas no significant differences were detected between the MS regions and either the HS or LS regions (**Figure 3B**).

Pearson’s correlation analysis between the OTUs and DI showed negative correlations in the HS regions (*r* = −0.35) and LS regions (*r* = −0.73), whereas the MS regions displayed a positive correlation (*r* = 0.30). In the infection samples, the HS regions still presented a weak negative correlation (*r* = −0.33), the MS regions presented a moderate positive correlation (*r* = 0.42), and the LS regions presented a weaker negative correlation (*r* = −0.38) (**Figure 3C**).

The Shannon diversity for both the infection and non-infection states was analyzed in relation to the DI, and the results revealed that, in the non-infection state, the HS regions showed a slight positive correlation (*r* = 0.27), whereas the MS regions exhibited a significantly negative correlation (*r* = −0.91, *p* = 0.034), indicating that greater fungal diversity in the non-infected samples was associated with lower disease severity in the MS regions. The LS regions displayed a weaker positive correlation (*r* = 0.40). In the infection state, the HS regions presented a moderate positive correlation (*r* = 0.74), suggesting that greater diversity in infected samples may correspond to greater disease severity. The MS regions showed a moderate negative correlation (*r* = −0.38), whereas the LS regions demonstrated a weak negative correlation (*r* = −0.18) (**Figure 3D**).

RDA revealed that under non-infected conditions, T and R were the primary environmental variables influencing community structure, with JN and ZZ showing unique microbial structures (**Figure 3E**). The overall variance explained by the first two axes (34.3%) suggested moderate environmental filtering of fungal endophytes in non-infected maize leaves (**Figure 3E**). In the infected group, T emerged as the dominant environmental factor, showing the longest gradient vector and strongest alignment with samples (LC and JN). In contrast, RH and R appeared to decrease, indicating that biotic stress may override the influence of microclimatic factors under pathogen pressure. DI exhibited a strong directional effect, suggesting that disease severity reshaped the endophytic community. The distinct spatial separation of samples along the RDA1 axis highlights the role of temperature and DI in driving microbial reassembly during SCR infection (**Figure 3F**).

### 3.4 Network analysis of the foliar endophytic community

**Figure 4:**
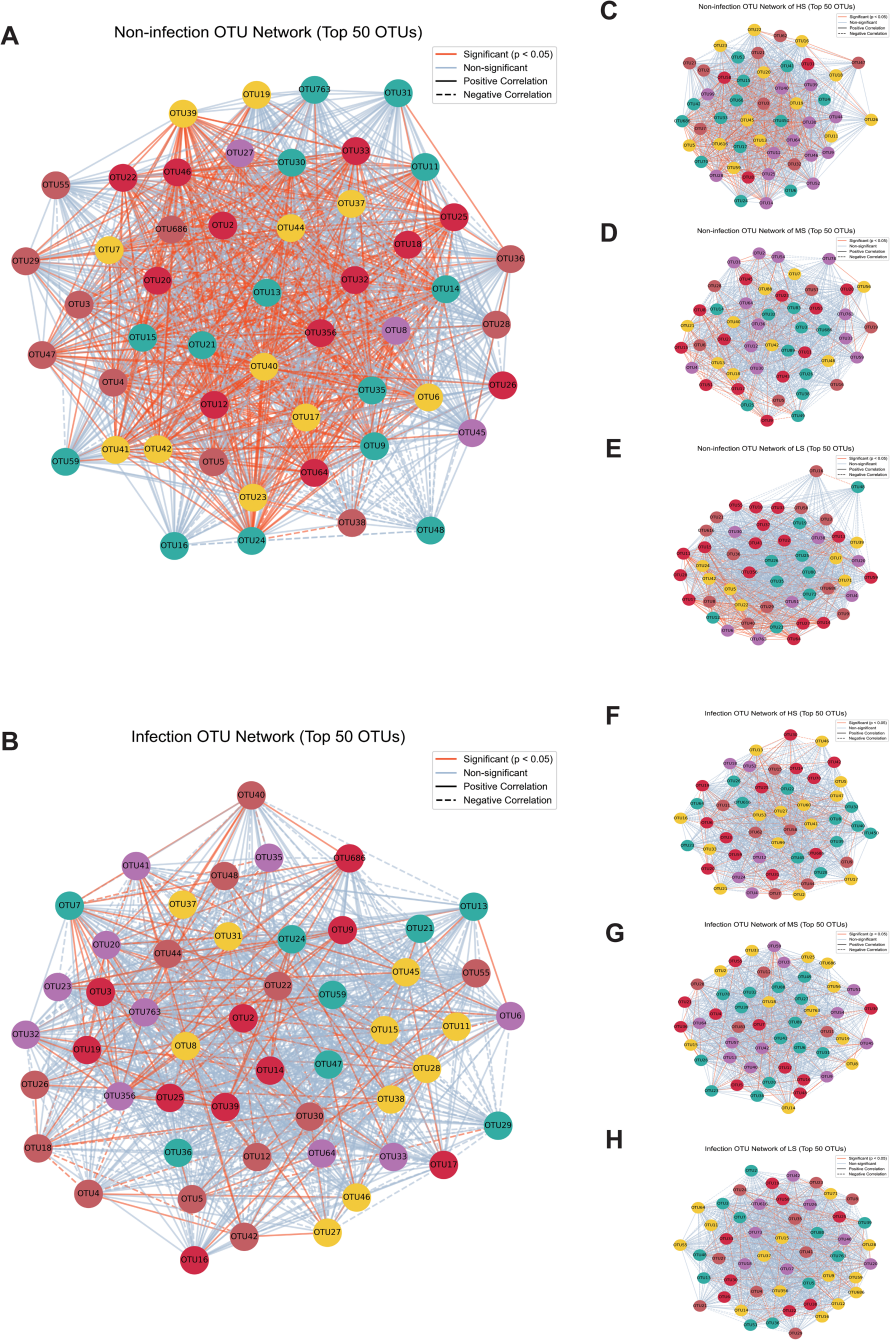
Microbial co-occurrence networks in infection and non-infection conditions across regions with high susceptibility (HS), middle susceptibility (MS), and low susceptibility (LS). **(A)** The microbial co-occurrence network under non-infection conditions. **(B)** The microbial co-occurrence network during *Puccinia polysora* infection. **(C, E, G)** The microbial co-occurrence network at the HS, MS, and LS regions under non-infection conditions. **(D, F, H)** The microbial co-occurrence network at the HS, MS, and LS regions under infection conditions. The orange lines indicate statistically significant correlations (*p* < 0.05), and the light blue lines represent non-significant correlations between operational taxonomic units (OTUs). The solid lines indicate positive correlations, and the dashed lines indicate negative correlations between OTUs.

Microbial co-occurrence networks were constructed to explore interactions among fungal genera under non-infected and infected conditions. The networks revealed distinct structural differences between the two groups. In non-infected samples, the endophytic fungal community exhibited a dense network with numerous significant positive correlations (*p* < 0.05), suggesting stable and cooperative interactions among endophytic taxa (**Figure 4A**). In contrast, the infection network showed a reduction in connectivity, with a higher proportion of non-significant correlations, indicating a disruption of microbial interactions under infection stress (**Figure 4B**).

Networks from the HS, MS, and LS regions further highlighted site-specific differences. Among the non-infected networks, the HS regions displayed the most highly connected network, followed by the MS and LS regions (**Figures 4C–E**). Upon infection, these networks exhibited reduced connectivity, with the HS regions maintaining higher connectivity than the MS and LS regions (**Figures 4F–H**). Some OTUs emerged as hubs-larger nodes with multiple connections, which are likely to represent key taxa that underpin network stability and functionality.

### 3.5 Correlation analysis between specific OTUs and the relative disease index

**Figure 5:**
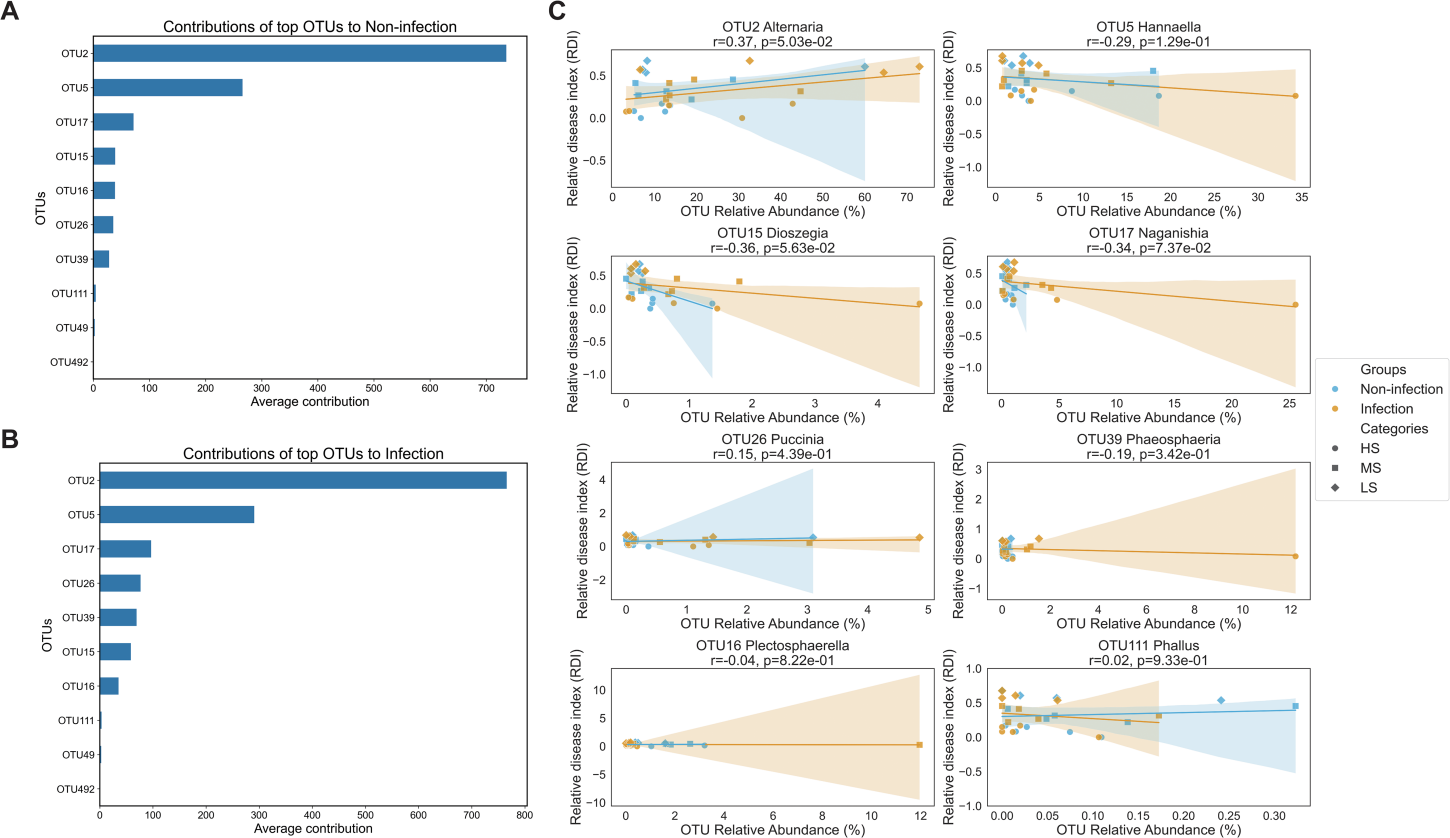
Functional prediction of foliar endophyte abundance and southern corn rust (SCR) severity. **(A, B)** Relative importance of the top 10 operational taxonomic units (OTUs) for predicting the severity of SCR as identified by random forest analysis, comparing the mean contribution values between the non-infected and infected groups. **(C)** Correlations between the relative abundances of eight high-prevalence fungal OTUs and the relative disease index (RDI) of the SCR associated with resistance. The symbols are colored according to the sampling site and shaped to indicate the SCR severity region categories (HS, MS, and LS). Each panel displays a linear regression line with a shaded area representing the 95% confidence interval. Pearson’s correlation coefficients (r) and corresponding *p*-values are provided for high-prevalence OTUs.

The random forest model revealed that OTU2 (*Alternaria*) and OTU5 (*Hannaella*) were the most influential taxa shaping community structure in both the non-infected and infected groups (**Figures 5A, B**). In the non-infected group, OTU15 (*Dioszegia*), OTU16 (*Plectosphaerella*), and OTU17 (*Naganishia*) were the main contributors to the microbial community structure (**Figure 5A**); in the infected group, OTU26 (*Puccinia*) and OTU39 (*Phaeosphaeria*) also emerged as major contributors (**Figure 5B**). These relationships, although moderate, were consistent across the sampling sites, suggesting a potentially conserved ecological role of these genera in SCR infection.

Correlation analyses revealed distinct association patterns between specific OTUs and RDI, where higher RDI values corresponded to increased resistance. Approximately 39% of the OTUs were associated with increased disease severity, whereas 61% were linked to potential roles in disease suppression. OTU2 was significantly positively correlated with RDI (*r* = 0.37, *p* = 0.05), suggesting its potential role in promoting host resistance to *P. polysora*. Conversely, OTU15 and OTU17 showed moderate negative correlations with RDI (*r* = −0.36, *p* = 0.056; and *r* = −0.34, *p* = 0.074, respectively), and the remaining OTUs exhibited weak or no significant associations (**Figure 5C**). Notably, the correlations tended to be stronger in the infected group than in the non-non-infected group, implying that microbial dynamics are more predictive of disease severity under pathogen pressure.

## 4 Discussion

### 4.1 Composition and diversity of foliar endophytes in maize

This study provides a comprehensive characterization of foliar fungal endophytic communities in maize under natural field conditions, comparing healthy (non-infected) and diseased (*P. polysora*-infected) states. Marked differences in community composition and relative abundance were observed between infection states, with variation also evident across geographically distinct sites. Genera such as *Alternaria*, *Blumeria*, *Hannaella*, *Naganishia*, and *Dioszegia* were consistently abundant across multiple sites, suggesting possible roles in disease progression or opportunistic colonization of infected tissues. Diversity analyses revealed that *P. polysora* infection significantly increased fungal richness but reduced community evenness, indicating a skewed proliferation of certain taxa in infected tissues, facilitated by pathogen-induced niche openings or host immune suppression. The PCoA results further supported the infection-driven restructuring of fungal communities, marked by an increased abundance of select genera, potentially contributing to disease progression and host stress responses. These findings emphasize the dual role of endophytic fungi during disease development, which act as mutualistic symbionts under healthy conditions while potentially transitioning into opportunistic pathogens under stress (Raghavendra and Newcombe, 2013; Abeywickrama et al., 2023; Zhang et al., 2018).

The regional patterns of foliar endophytic fungal richness followed a trend of LS < MS < HS, with a 1.3-fold increase observed between the LS and HS regions. This difference suggests that local foliar endophytes change in relation to the severity of the disease. Fungal richness was significantly greater in *P. polysora*-infected samples from the HS regions than in those from the LS regions, suggesting pathogen-driven community expansion. However, the correlation between OTU richness and DI showed divergent patterns across susceptibility levels, with the LS regions showing a strong negative trend (*r* = −0.73), although the difference was not statistically significant, likely due to the limited sample size. These findings support the hypothesis that fungal diversity increases with disease progression (Wagner et al., 2020; Martins et al., 2021) and highlight the importance of site-specific microbial dynamics in shaping host–pathogen interactions. These results underscore the critical role of foliar endophytes in SCR infections and plant stress responses, providing a foundation for future investigations into their potential applications in disease prevention and management strategies.

### 4.2 Network analysis of microbial interactions

Co-occurrence network analysis revealed core fungal genera shared among multiple study sites, suggesting that the presence of conserved taxa is critical for maintaining host health and mediating responses to *P. polysora* infection, revealing the profound effects of *P. polysora* infection on community stability and diversity. Under non-infected conditions, microbial networks were dense and cooperative, reflecting the stability and connectivity of endophytic fungal communities in healthy hosts. The microbial networks from the HS regions presented the highest connectivity, followed by those from the MS and LS regions, indicating site-specific differences in network complexity. Upon infection, network connectivity declined significantly, characterized by fewer significant correlations and fragmented networks. This destabilization was particularly evident in the LS and MS regions, whereas the HS regions retained greater connectivity, suggesting that certain endophytic fungal communities exhibit greater resistance to infection. The shifts in endophytic fungal diversity and network stability observed in response to *P. polysora* infection are broadly consistent with previous reports in other pathosystems (Wagner et al., 2020; Ampt et al., 2022), such as corn leaf blight in maize (Chao et al., 2025), *Melampsora* rust in poplar (Raghavendra and Newcombe, 2013), and powdery mildew in pumpkin (Zhang et al., 2018). This shift toward a more heterogeneous and unstable microbial network could compromise the functional resilience of the microbiome, thereby influencing disease progression and plant fitness. This finding suggests that infection-driven perturbations reshape endophytic community dynamics and highlight keystone taxa as potential targets for biocontrol interventions aimed at disease suppression or mitigation.

### 4.3 Associations of specific fungal genera with SCR susceptibility

Keystone taxa emerged as critical nodes within microbial networks, maintaining community structure and functionality. The infection-induced loss or diminished connectivity of these keystone taxa underscores their importance in sustaining microbial stability (Ampt et al., 2019, 2022). Our findings demonstrate that specific fungal genes are distinctly associated with SCR severity, highlighting the functional divergence of endophytic fungal communities in non-infected and infected states. *Alternaria* emerged as a dominant genus in both healthy and infected tissues, and its positive correlation with disease severity suggests that it may act as a latent pathogen or disease-facilitating endophyte. This dual role aligns with previous observations (Busby et al., 2016), where *Alternaria* enriched under stress may serve as a competitive colonizer or conditionally pathogenic taxon. Furthermore, 60% of the genera were identified as potential protective agents, likely modulating host resistance to *P. polysora*, underscoring their potential as biocontrol agents for targeted disease management. The core genera identified here have been consistently reported in maize studies across diverse environments (Johnston-Monje and Raizada, 2011; Busby et al., 2016; Chao et al., 2025), suggesting a degree of ecological generalizability. These data support the concept of disease-modulating endophytic consortia and emphasize the need for functional validation of candidate taxa. While further validation is needed, the selective enrichment of beneficial endophytes through microbial inoculants or cropping practices could increase maize resilience.

### 4.4 Environmental and geographic factors shaping endophytic communities

Our results also revealed that shifts in foliar endophytic communities in response to *P. polysora* infection were modulated by environmental and geographic factors. Regional variation in pathogen pressure, along with local microclimatic conditions such as temperature and humidity, contributed to the observed differences in both disease severity and microbial community composition. These results suggest that pathogen stress amplifies the influence of environmental variables on endophyte assembly, driving spatial heterogeneity in community dynamics and disease outcomes. Although sampling was standardized at the grain-filling stage, unaccounted seasonal and weather-related fluctuations may further shape the microbial structure and confound infection effects. These findings emphasize the importance of incorporating site-specific environmental variables into predictive models of SCR risk and microbiome dynamics in future research. Such models could facilitate the disentanglement of the direct effects of endophytic communities on SCR progression from the indirect effects of environmental drivers.

## 5 Conclusions

This study highlights the critical role of foliar endophytic community restructuring in mediating maize resistance to SCR under natural environmental conditions. By linking changes in SCR severity to shifts in foliar endophytic communities, we provide novel insights into the maize–*P. polysora* pathosystem. Core fungal genera such as *Alternaria* and *Kodamaea* have emerged as key modulators of disease severity, whereas *Hannaella* and *Naganishia* have been implicated in disease facilitation, offering a nuanced understanding of the dual roles of endophytes in host–pathogen interactions. The identification of fungal taxa with potential biocontrol activity presents opportunities for the development of sustainable disease management strategies. Future studies should incorporate multi-seasonal sampling, controlled inoculation experiments, and detailed microclimatic monitoring to better understand the ecological mechanisms shaping the foliar microbiomes of maize. Functional validation of candidate taxa through culture-dependent methods and *in planta* assays will be critical for confirming their biocontrol potential. Additionally, predictive models incorporating host–microbiome interactions under varying environmental conditions could offer critical insights into endophyte-mediated plant defense mechanisms. Such efforts will facilitate the development of targeted biocontrol strategies that leverage the natural variability in fungal communities to mitigate the severity of SCR and increase crop resilience.

## Data Availability

The original contributions presented in the study are publicly available. This data can be found here: NGDC, PRJCA040135 https://ngdc.cncb.ac.cn/gsa.

## Supplementary material

Supplementary Figure 1 The relative abundance of fungal species across different sampling sites under infection and non-infection states. The size of the bubbles indicates the relative abundance of each species, and the color gradient reflects their abundance levels, with yellow representing higher abundance and blue representing lower abundance.
