# Supplementary figures and images for: The role of foliar endophytes in modulating southern corn rust severity: implications for biocontrol strategies
Source: Front Plant Sci. 2025 May 23;16:1554915. doi: 10.3389/fpls.2025.1554915 (PMC12141246; doi:10.3389/fpls.2025.1554915)

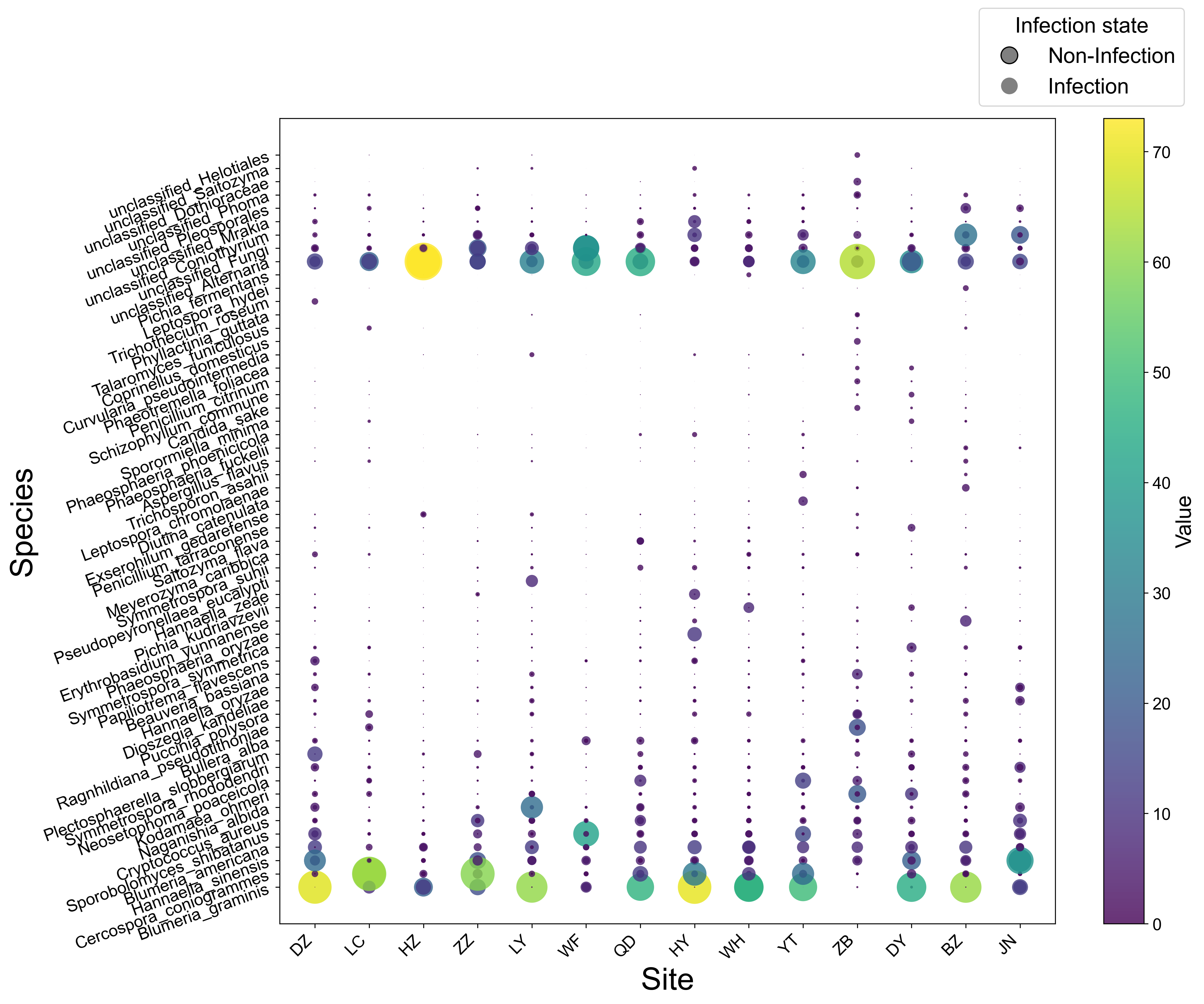

Supplement: Supplementary Figure 1 — The relative abundance of fungal species across different sampling sites under infection and non-infection states. The size of the bubbles indicates the relative abundance of each species, and the color gradient reflects their abundance levels, with yellow representing higher abundance and blue representing lower abundance. [file Image1.png]
